# The added value of three-dimensional transthoracic echocardiography in mitral annular disjunction: a case report

**DOI:** 10.3389/fcvm.2024.1366444

**Published:** 2024-03-27

**Authors:** Konstantinos Papadopoulos, Ignatios Ikonomidis, Mani A. Vannan

**Affiliations:** ^1^ Echocardiography Laboratory, European Interbalkan Medical Center, Thessaloniki, Greece; ^2^ Echocardiography Laboratory, 2nd Cardiology Department, Medical School, Attikon University Hospital, National and Kapodistrian University of Athens, Athens, Greece; ^3^ Structural and Valvular Center of Excellence, Marcus Heart Valve Center, Piedmont Heart Institute, Atlanta, GA, United States

**Keywords:** MAD syndrome, 4D strain, 3D echocardiography, arrhythmic mitral valve prolapse, case report

## Abstract

**Background:**

Mitral annular disjunction (MAD) refers to the arrhythmic mitral valve prolapse (MVP) syndrome associated with ventricular arrhythmias and sudden cardiac death. Although the pathophysiology of this disease is still under investigation, specific imaging criteria that establish the diagnosis have been recognized. In this article, we demonstrate most of these criteria using three-dimensional transthoracic echocardiography (3D-TTE) and provide added value in the management of MAD syndrome.

**Case presentation:**

A 50-year-old male patient with recent syncope and a history of mitral regurgitation (MR) and MAD was admitted to our clinic for further investigation. According to our protocol, the patient underwent a complete 3D-TTE, laboratory blood exams, and 24 h ambulatory electrocardiogram (ECG). Our investigation confirmed the presence of MAD syndrome with bileaflet prolapse, severe MR, and non-sustained ventricular tachycardia, necessitating an implantable cardioverter defibrillator (ICD) and surgical mitral valve repair. The 3D-TTE analysis of the mitral valve demonstrated mitral annular systolic expansion and systolic flattening of the saddle-shaped annulus and quantified the extent of the disjunction arc. Additionally, four-dimensional (4D) strain analysis of the left ventricle revealed the presence of fibrosis of the posteromedial papillary muscle and basal inferolateral wall, which are variables that are required for the diagnosis and therapeutic management of MAD syndrome.

**Conclusions:**

3D-TTE and 4D strain offer valuable insights for diagnosing and managing patients with MAD syndrome. This method seems to correlate well with the other imaging modalities and could be included in the management protocol of MAD syndrome.

## Introduction

MVP is one of the most common valvulopathies with a normally benign course. It affects 2%–3% of the general population and surgical repair is the gold standard treatment (1–3). Less than 1% of the patients with MVP present with malignant arrhythmias and experience sudden cardiac death (4, 5). Extent research from autopsies has recognized an entity that correlates with ventricular arrhythmias called mitral annular disjunction (MAD). MAD is characterized by the separation of the hinge point of the posterior mitral leaflet from the posterior ventricular wall, systolic expansion and flattening of the annulus, curling motion of the basal inferolateral left ventricular (LV) wall, and the presence of segmental fibrosis of the posterior wall or the posteromedial papillary muscle (PM) (6, 7). The standard echocardiographic approach starts with a parasternal long-axis view for evaluation of the aforementioned findings. A distance of the posterior leaflet from the LV wall of >5 mm is considered pathognomonic for the presence of MAD. Typically, patients with MAD have Barlow's disease or bileaflet prolapse with myxomatous degeneration. Further TTE analysis should include tissue Doppler imaging for the “Pickelhaube” sign (high-velocity spike of the lateral mitral annulus, >16 cm/sec) and speckle tracking analysis for assessment of the mechanical dispersion of the LV (the standard deviation of contraction duration of LV segments, normal median values = 21 msec) (8, 9).

The gold standard method for diagnosing MAD syndrome is cardiac magnetic resonance imaging (CMR) due to its higher spatial resolution capable of detecting MAD of even >2 mm. Additionally, it can confirm the presence of fibrosis and scarring through late gadolinium enhancement (LGE) (10, 11). Echocardiography remains the first-line diagnostic method for patients with MVP. Several publications have already described the imaging findings of MAD using 3D transesophageal echocardiography (TEE), computed tomography (CT), and CMR (12–14). However, since 3D-TTE is less invasive than TEE and less costly than CMR and cardiac CT, it can be considered the preferred method when feasible. This study aims to give the methodology of how to evaluate the dynamic changes of mitral annulus in MAD syndrome with 3D-TTE and search for myocardial fibrosis with 4D strain. To our knowledge, this is the first described MAD case in the literature evaluated using 3D-TTE.

## Case presentation

### Patient clinical and echocardiographic characteristics

**Figure 1:**
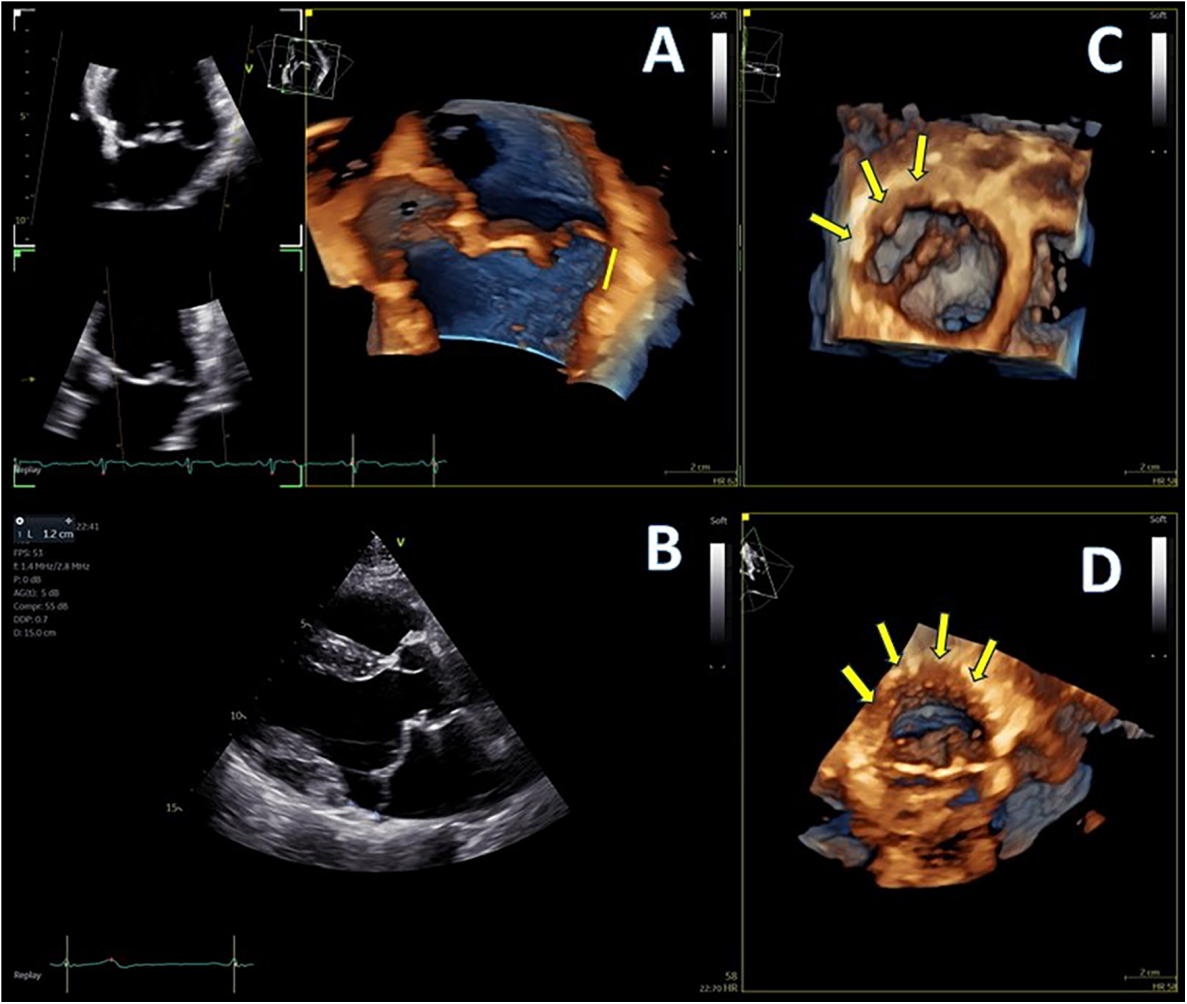
(**A**,**B**) Mitral annular disjunction (MAD) demonstrated by 4D and 2D transthoracic echocardiography (TTE), 12 mm of distance from posterior left ventricular (LV) wall, indicated with yellow line, and (**C**,**D**) extent of disjunction arc seen with two different orthogonal volume rendering views, indicated with yellow arrows.

**Figure 2:**
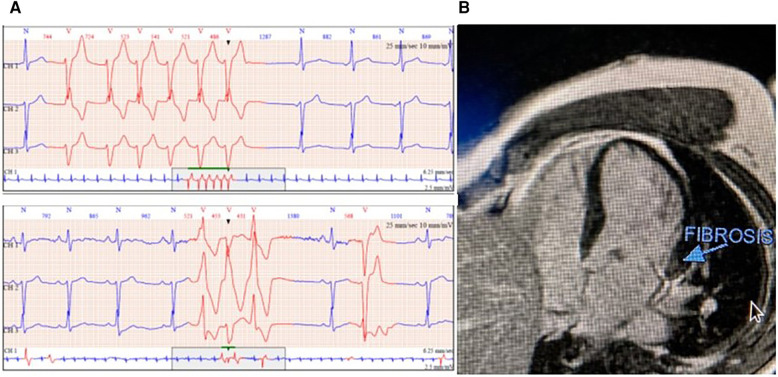
(**A**) Monomorphic and polymorphic ventricular tachycardia in 24 h ambulatory electrocardiogram (ECG) and (**B**) cardiac magnetic resonance imaging (CMR) with fibrosis detection of the basal inferolateral wall.

A 50-year-old male patient with mitral regurgitation (MR) and MAD previously diagnosed with CMR was referred to our clinic due to a recent syncopial episode. TTE revealed severely dilated left heart chambers (4D left ventricular end-diastolic volume (LVEDV) = 218 ml, left atrial volume index (LAVI) >48 ml/m^2^) with an LVEF of 57%, bileaflet prolapse of the mitral valve, severe MR [effective regurgitant orifice area (EROA) 60 mm^2^, regurgitant volume (RV) 117 ml], and a 12 mm displacement of the hinge point of the mitral annulus from the posterior ventricular wall (Figures 1A,B). Further complete 3D-TTE analysis of the patient was performed to demonstrate the imaging findings of MAD syndrome (Figures 1C,D). A previous CMR examination of the patient had reported LV dilatation with almost identical volumes and EF to TTE (EDV = 217 ml, EF = 59%), the presence of MAD of 10 mm displacement, and the presence of myocardial fibrosis of the basal inferolateral wall (Figure 2). Due to our protocol for patients with syncope, we performed a 24 h ambulatory electrocardiogram (ECG) that revealed episodes of monomorphic and polymorphic non-sustained ventricular tachycardia (Figure 2). In accordance with the management algorithm of the recently published EHRA and EACVI consensus document for MAD syndrome (15), the patient was treated with surgical mitral valve repair and implantable cardioverter defibrillator (ICD) implantation.

### 3D transthoracic echocardiography analysis

The 3D-TTE examination was performed with the GE Vivid E95 echo machine (GE Vingmed Ultrasound, Horten, Norway), using the 4Vc probe, and all data were stored in the EchoPAC v.203 workstation.

**Figure 3:**
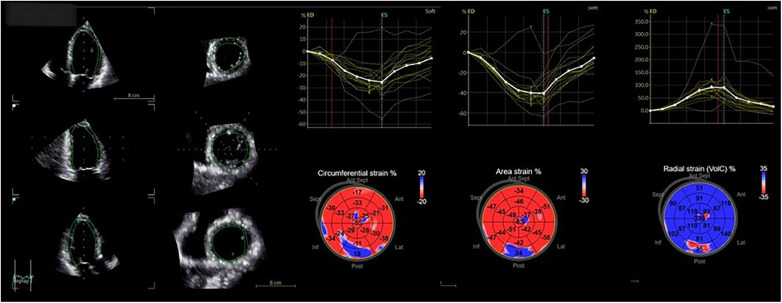
Four-dimensional strain analysis showing abnormal values of circumferential, area, and radial strain of the basal inferolateral wall.

Advanced LV assessment and 4D strain analysis values were performed with the dedicated AutoLVQ application, using a full-volume apical four-chamber view, focused on the LV, with a frame rate of >25 vps. Global LV strain values were overall normal. The longitudinal strain was measured at −21%, circumferential strain at −20%, radial strain at 69%, and area strain at −40%. The strain values of the basal inferolateral segment were reduced, confirming the presence of the fibrosis detected in the MRI examination of the patient. The segmental radial strain was measured at 35% (normal values at 43.2 ± 4.5%), area strain at 24% (−36.5 ± 3.9%) (with adjacent segmental values of −47 and −56%), and circumferential strain at 18% (−30.3 ± 4.0%), while the longitudinal strain was preserved at −22% (−21 ± 0.6%) due to the hyperdynamic motion of the basal posterior wall (16) (Figure 3).

**Figure 4:**
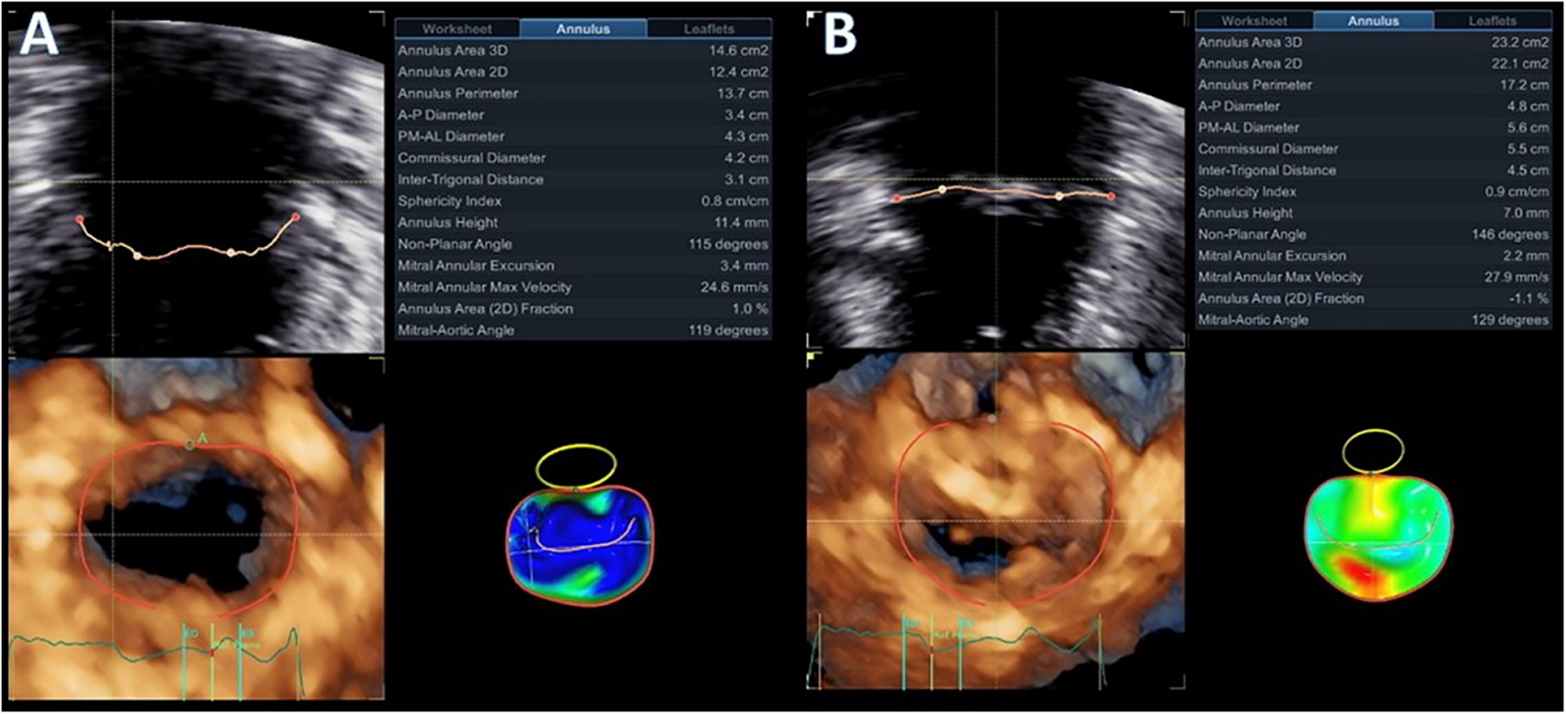
Diastolic (**A**) and systolic (**B**) mitral valve quantification (MVQ) models showing the dynamic changes and the systolic expansion and flattening of the mitral annulus.

Advanced mitral valve assessment was performed with the AutoMVQ method that provided the measurements of the leaflets and the annulus, using an apical four-chamber view, focused on the mitral valve with 4D zoom and a frame rate of >12 vps. By default, AutoMVQ creates the mitral valve model using a middle reference frame between end-diastole and end-systole. Since the requested information for MAD syndrome is the dimension changes between diastole and systole, we had to manually adjust the systolic and diastolic frames twice and provide two different mitral valve quantification (MVQ) models. A direct comparison of the systolic and diastolic models allows us to understand the dynamic annulus changes in MAD syndrome. In our patient, this method confirmed (1) the flattening of the saddle-shaped annulus during the systole and (2) the systolic expansion. In particular, the annulus area increased from 14.6 cm^2^ to 23.2 cm^2^, the perimeter from 13.7 cm to 17.2 cm, the anteroposterior diameter from 3.4 cm to 4.8 cm, and the commissural diameter from 4.2 cm to 5.5 cm. Meanwhile, the annulus height decreased from 11.4 cm to 7.0 cm (Figure 4). The disjunction arc of the valve was easily demonstrated by an “en face” 3D ventricular view of the mitral valve where the extent of the arc was assessed by measuring the circumference of the posterior annulus involved within the disjunction. Volume rendering views can reveal the actual part of the free posterior wall of the left ventricle that is separated from the annulus and the atrium (Figures 1C,D).

## Discussion

Recognition of MAD syndrome with myocardial fibrosis is of major importance, as it has been correlated with malignant arrhythmias and sudden cardiac death (4, 17). Patients with myxomatous mitral valve disease should be investigated for the presence of MAD (18). This information is also important when planning surgical repair for such patients, as the surgeon may need to use longer stitches and stabilize the annulus ring close to the posterior wall of the left ventricle (19, 20). The advantage of CMR with its high spatial resolution is the ability to simultaneously assess the morphology and the dynamics of the mitral valve, the volumes and performance of the left ventricle, and the presence of myocardial fibrosis (12, 13, 21). If it is feasible to appreciate most of these variables with the less invasive and costly 3D-TTE, then CMR might not be the first-line method in the future.

In this MAD case, we have presented the methodology of how to evaluate the dimensions and the dynamic changes of the mitral annulus with 3D-TTE. The MVQ method is widely recognized providing detailed information on the dimensions of the mitral valve, which correlates well with cardiac CT and has already proven its value in the management of MR (6, 22). 3D-TTE and 3D-TEE also correlate well (23), making transthoracic echocardiography reliable for giving accurate MVQ measurements. Previous published studies have only included MAD patients evaluated by TEE. Since our routine practice stands on transthoracic echocardiography and we aim to provide diagnosis with the minimum radiation exposure (instead of cardiac CT), cost (instead of CMR), and minimally invasive way (instead of TEE), 3D-TTE emerges as the most attractive alternative method. In our case, 3D-TTE was able to demonstrate most of the required imaging characteristics of MAD syndrome.

A standard 2D parasternal long-axis view was the first image that was used to confirm the presence of MAD syndrome by the “uncoupling” of the posterior ventricular wall from the hinge point of the posterior mitral annulus. A distance of more than 5 mm is usually considered to be diagnostic for the presence of MAD (14). 3D-TTE was able to provide detailed information about the presence and extent of the disjunction arc, the systolic expansion, and the flattening of the saddle-shaped annulus. The extent of the disjunction arc correlates with the severity of MR and the abnormal dynamics of the valve (6). It is well established from previous publications (6, 13, 24) that at the end-diastole, the annulus dimensions are at their maximum, with the anteroposterior diameter and annulus area presenting the greater change. Physiologically, the annulus contracts rapidly at the early systole and then starts to expand. In normal subjects, the end-systolic annulus area is smaller than the end-diastolic one (6). Patients with myxomatous valve disease have larger annulus dimensions, the valve expands even more at the late-systolic period, but the overall annulus area does not reach the end-diastolic measurements (6, 25). In MAD syndrome, on the other hand, the baseline dimensions of the annulus are increased. There is still early systolic contraction of the valve, but, afterward, there is overexpansion of the annulus, with dimensions that exceed the diastolic ones (25). Manual adjustments of the standard MVQ methodology were able to appreciate the systolic expansion and flattening of the annulus by measuring all annulus dimensions and height changes throughout the cardiac cycle. The two frames that we used were the end-diastolic and end-systolic ones to be in agreement with previous publications that analyzed the annulus dynamics.

The 4D strain method was further used to give information about the presence of ventricular myocardial fibrosis and was included in the management algorithm of the patient (26). Segmental basal inferolateral wall strain values were significantly reduced especially the area strain which is a parameter that can be evaluated only with 4D strain. Since in MAD there is segmental hypercontractility of the basal inferolateral wall, 2D strain/speckle tracking evaluation of longitudinal strain is of limited value as longitudinal deformation is increased in this part of the LV and radial and circumferential 2D strain values are not reproducible since it is easy to make errors due to variable/off axis plane selection and through-plane motion (27). 4D strain, on the other hand, can simultaneously provide measurements for longitudinal, radial, circumferential, and area strain. In our patient, except for the longitudinal strain, which was unaffected, the presence of fibrosis and the systolic stretching of the basal inferolateral wall resulted in the reduction of the radial strain values and in positive values of circumferential and area strain that confirmed the presence of fibrosis and the lengthening of the fibers during systole due to the disjunction arc. From this point of view, 4D strain seems superior and may be the only echocardiographic method that can detect myocardial abnormalities in MAD cases. A limitation of this method that probably still makes CMR the gold standard diagnostic tool is the inability to detect fibrotic tissue on the posteromedial PM. Strain analysis by default excludes papillary muscles from tracking, and it is impossible with the present applications of 4D strain to extend our analysis to the PM.

## Limitations

It has to be mentioned that this specific patient had excellent 2D and 3D images, which made possible all this extensive analysis of the annulus dimensions and dynamics. This method might have unreliable results in the case of suboptimal imaging and low frame rate acquisitions. It is common knowledge that we cannot provide reliable 3D volume-rendered images without decent baseline 2D images. Frame rate, on the other hand, is also very important since we need to determine precisely the true end-diastolic and end-systolic frame. Frame rate furthermore affects the 4D strain analysis. For accurate results, we need >25 volumes per second (vps), but this number is even quite low for information like mechanical dispersion. Mechanical dispersion analysis requires >50 vps; otherwise, the measurements in milliseconds will not be accurate. Currently, with the provided technology it is difficult to acquire full-volume images of the left ventricle, including the epicardial layer, with high frame rates. Next-generation vendors and the evolvement of the probes may overcome this issue.

Another restriction of this approach is the time that is needed for this extensive analysis. Previous publications have shown that 3D-TTE is a time-saver (28) and does not necessarily require big experience. However, the imager should be familiar with the MVQ application and 4D strain analysis in order to be able to include this approach in routine practice. Since MAD is not present in everyday practice though, in our opinion, it is worth it to spend some time on this detailed 3D analysis.

## Conclusions

The MAD syndrome is not yet fully investigated. The first important step for the management of these patients is correct diagnosis. A complete 2D/3D-TTE protocol seems to be able to describe most of the required imaging features of this disease and detect the presence of fibrosis. This is the first MAD case in the literature, analyzed with 3D-TTE with results confirmed with CMR. Even though our initial experience is promising, this remains one single case reported and further investigation should be done to validate the method and routinely include 3D-TTE in our diagnostic protocols.

## Data Availability

The raw data supporting the conclusions of this article will be made available by the authors, without undue reservation.
